# Direct detection of circulating donor-derived extracellular vesicles in kidney transplant recipients

**DOI:** 10.1038/s41598-022-26580-6

**Published:** 2022-12-20

**Authors:** Wouter W. Woud, Dennis A. Hesselink, Martin J. Hoogduijn, Carla C. Baan, Karin Boer

**Affiliations:** Division of Nephrology and Transplantation, Department of Internal Medicine, Erasmus MC Transplant Institute, Erasmus University Medical Center, Molewaterplein 40, 3015 GD Rotterdam, The Netherlands

**Keywords:** Nanoparticles, Flow cytometry, High-throughput screening

## Abstract

Extracellular vesicles (EVs) are tissue-specific particles containing valuable diagnostic information. However, single EV analysis in blood is challenging due to their physical properties, the molecular complexity of plasma, and a lack of robust data interpretation methods. We assess the applicability of our recently-developed calibrated Imaging Flow Cytometry (IFCM)-based methodology to detect/characterize circulating tissue-specific EV subsets in the clinical setting of kidney transplantation. Platelet-poor plasma was generated from 36 HLA-A3 mismatched donor (HLA-A3 +) and kidney transplant recipients (KTRs; HLA-A3-). Samples taken before transplantation, 3 days, 7 days, and 6 months after transplantation as well as before ‘for-cause’ kidney transplant biopsies were stained with anti-CD9 (plasma EV-marker) and anti-HLA-A3. Before transplantation, no significant differences in total CD9 + EV concentrations were detected between donor and KTR samples. Tissue-specific EVs were identified as CD9 + HLA-A3 + . Serial dilution experiments of HLA-A3 + in HLA-A3- PPP showed that single CD9 + HLA-A3 + EVs were detectable down to ~ 1% above the recipient ‘self-signal’. After transplantation, CD9 + HLA-A3 + EVs were detected above pre-transplantation concentrations in individuals with stable allograft function, but not in individuals with allograft dysfunction. These results demonstrate the applicability of our calibrated IFCM-based methodology in the direct detection of tissue-specific EV subsets in clinical samples. We believe that this EV methodology is applicable in a variety of clinical contexts.

## Introduction

Extracellular vesicles (EVs) are lipid bilayer-delimited membrane particles (30–8000 nm in diameter^1^) excreted by all cell types, which act as signaling intermediaries during normal homeostasis and during pathologic processes^2–6^. EVs carry proteins on their surface and/or a variety of macromolecules as cargo (e.g. DNA, RNA, lipids and proteins^7^), which are thought to reflect the status of their cell of origin. Indeed, EVs are regarded as “snapshots” of the status of their cell of origin and are examined to assess the presence of various diseases, e.g., cancer or viral infection^8,9^. As EVs are present in body fluids (e.g. blood^1^/saliva^10^/urine^11,12^), they are considered to be minimally-invasive biomarkers and so-called "liquid biopsies"^2,13–16^.

The quantification and characterization of EVs is hampered by their physical characteristics such as their small size, low epitope copy number^17^, and the variety of protein markers depending on the cell source^18,19^, all of which contribute to the well-documented EV heterogeneity. The identification of EVs in plasma is further hindered by the molecular complexity of the plasma, which contains multiple elements (e.g., lipoproteins, cell debris and soluble proteins), that interfere with EV analysis^18,20^. Moreover, a lack of robust methods and ambiguities in how data should be interpreted for EV analysis, makes comparison between studies challenging^21,22^.

To overcome the selection biases or introduction of artefacts that are introduced by performing commonly used EV isolation methods^18,20^, our group recently developed a standardized Imaging Flow Cytometry (IFCM)-based methodology for the direct measurement of single EVs ≤ 400 nm in diameter in diluted plasma samples, *without* prior isolation of EVs^23^. By omitting the need for sample isolation, this method has the potential to directly show the status of an individual by measuring distinct EV subsets, which is greatly beneficial for the monitoring of EVs in health and disease^21,22^.

Expanding upon the previously reported ability of our methodology to directly detect and discriminate human- and mouse-derived EVs in mixed human/mouse plasma samples^23^, we here aimed to assess the ability of our protocol to identify, discriminate and analyze EV subsets within human patient plasma samples. To this end, the setting of clinical organ transplantation offers a unique scenario in which tissue-specific EVs originating from the allograft are released into circulation after transplantation^14,24,25^. We present an application of our methodology that allows for the direct detection of donor-derived EVs (dd-EVs) in plasma samples from kidney transplant recipients (KTRs) on the basis of donor-recipient Human Leukocyte Antigen (HLA) mismatching by analyzing samples obtained before (control, no donor EVs present) and after (signal validation, detection of dd-EVs originating from the allograft) transplantation. Dd-EVs were measured against a background of recipient EVs in longitudinally collected KTR samples, and the dynamics of dd-EVs was monitored over time, as well as at time of ‘for-cause’ biopsy samples.

## Results

### Direct detection of single EVs ≤ 400 nm in donor and KTR plasma samples

Compared to donor samples, the plasma composition of KTRs contains multiple elements (e.g., elevated creatinine and urea, (medicinal) waste products) which may interfere with single EV detection. Therefore, we first tested the ability of our protocol to directly detect single EVs ≤ 400 nm in diameter in plasma samples obtained from KTRs before transplantation. To this end, PPP samples from living donors (used as healthy controls) and KTRs were diluted in fPBS, and stained with either anti-CD9 (as common plasma EV marker) or isotype control (to infer labelling specificity) before analysis with IFCM. After initial acquisition, detergent treatment was applied on each anti-CD9 labeled sample to discriminate between vesicular and non-vesicular events.

**Figure 1:**
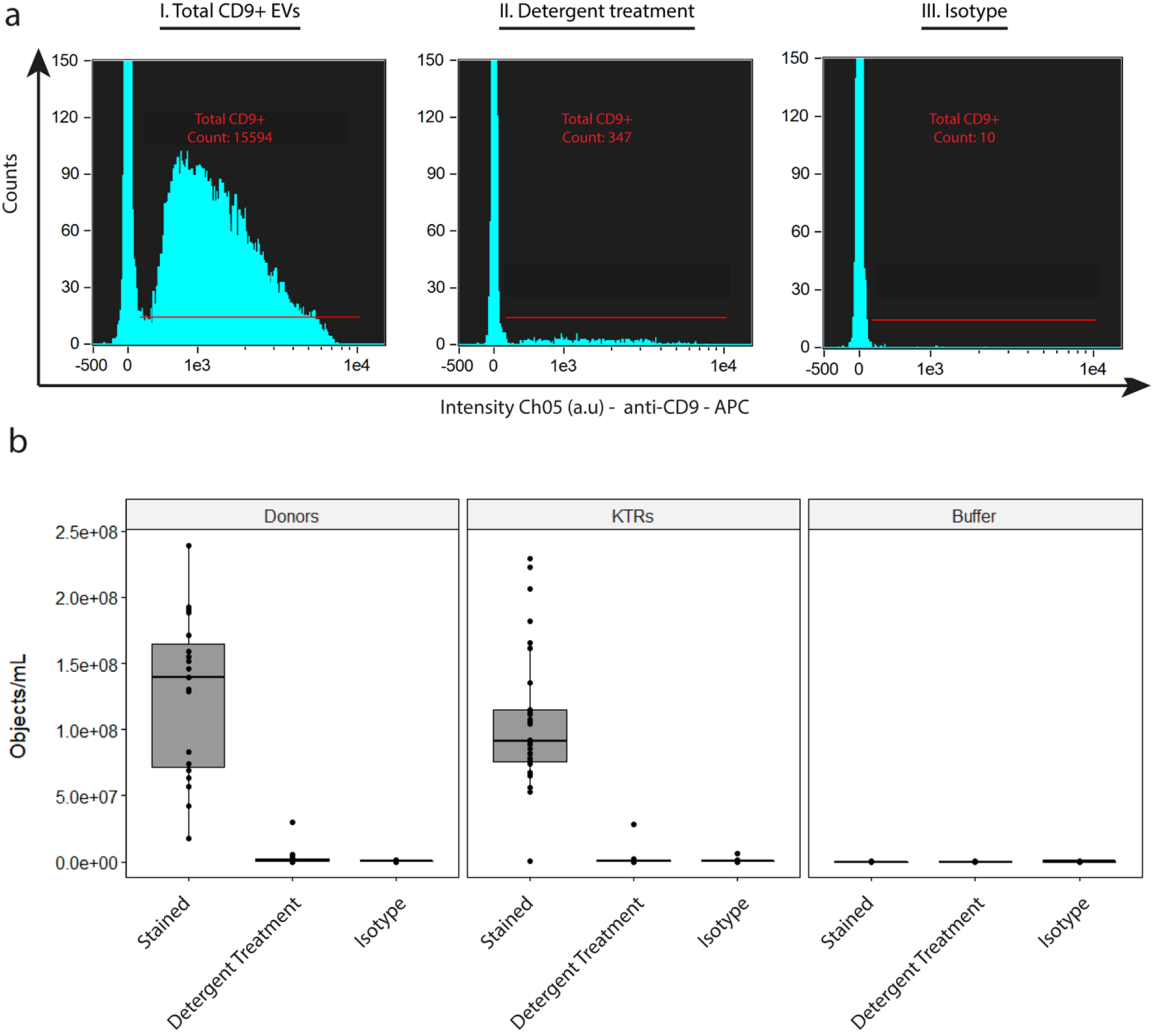
Detection of CD9 + EVs in both (living) donor and kidney transplant recipient (KTR) samples obtained before transplantation. (**a**) left to right: representative histogram plots of a PPP sample stained with anti-CD9, the same sample after detergent treatment (to discriminate between vesicular and non-vesicular events), and isotype staining. (**b**) For each of the controls applied: quantification of total CD9 + EV concentrations as detected in PPP samples obtained from (living) donors, KTRs, nd buffer only controls.

Total concentrations of CD9 + objects/mL (Fig. 1a) as measured in donor and KTR plasma samples were compared (Fig. 1b). In the donor group, we detected 1.26E^8^ ± 6E^7^ objects/mL before, and 2.74E^6^ ± 6.7E^6^ objects/mL after detergent treatment. This represented an approximate 98% reduction—which implies that the majority of CD9 + events analyzed represent EVs. In the plasma samples obtained from KTRs, we detected 1.07E^8^ ± 5.3E^7^ objects/mL before, and 1.6E^6^ ± 5.1E^6^ objects/mL after detergent treatment (~ 97% reduction). No significant differences in total CD9 + EV concentrations were observed between both groups. Additionally, isotype staining resulted in 4.16E^5^ ± 3.85E^5^ objects/mL and 5.27E^5^ ± 1.22E^5^ objects/mL for donor and KTR samples, respectively, illustrating the high specificity of our labelling strategy (~ 300-fold difference with mAb labelling). Buffer only controls (measured each day of sample acquisition) showed no detectable events – indicating that no signals derived from antibody aggregates were detected.

These data suggest that our IFCM protocol is able to directly detect single CD9 + EVs in diluted plasma samples obtained from donors and KTRs before transplantation, despite the differences in molecular composition of the plasma between both groups.

### Identification and discrimination of single EVs on the basis of HLA phenotype

In order to identify dd-EVs in the circulation of KTRs after kidney transplantation, we assessed whether IFCM is capable of discriminating EVs based on HLA phenotype. To this end, we labelled the same PPP samples (donors—HLA-A3 + , and recipients (before KTx)—HLA-A3 − ) with anti-CD9 and anti-HLA-A3, and performed the same control experiments as described above.

**Figure 2:**
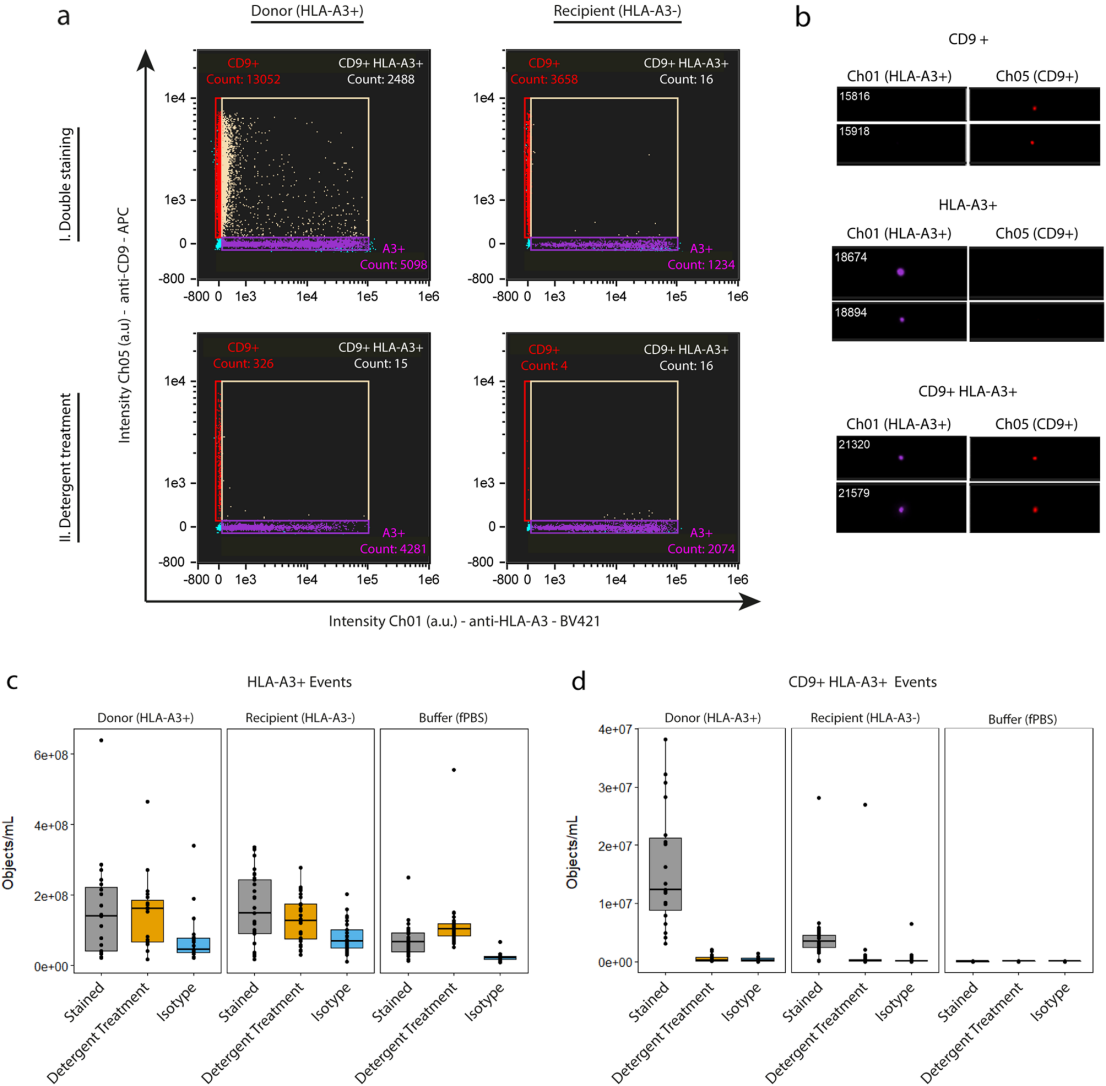
Discrimination of EVs based on HLA phenotype. (**a**) Representative scatter plots of an HLA-A3 + PPP sample (left) and an HLA-A3- PPP sample (right) stained with anti-HLA-A3 (X-axis) and anti-CD9-APC (Y-axis) before and after detergent treatment (top and bottom row, respectively). (**b**) Visual examination of events representative for each fluorescent population demonstrated the selection and subsequent analysis of particles showing single spot fluorescence (no coincidence events), indicating the selection and analysis of single EVs. (**c**,**d**) Concentrations of HLA-A3 + and CD9 + HLA-A3 + fluorescent events in all pre-transplantation donor, recipient, and buffer only (control) samples before and after detergent treatment, and after isotype staining (grey, orange, and blue boxes, respectively). HLA-A3 + events were found to not be indicative of EV signals and were excluded from analysis. Double-positive events were found to be indicative of EV signals, as well as to be discriminative between HLA-A3 + and HLA-A3- PPP samples.

Figure 2a shows representative scatter plots for both a donor and recipient PPP sample analyzed with IFCM, before and after detergent treatment. Visual examination of events representative for each fluorescent population (before detergent treatment) demonstrated the selection and analysis of particles showing single spot fluorescence (no coincidence events), indicating the selection and analysis of single EVs^23^ (Fig. 2b).

For HLA-A3 + single-positive events, we observed only marginal reduction by detergent treatment, showing that these events are not representative of biological particles. Moreover, buffer only controls showed a high degree of fluorescent events, indicating that these signals are most likely representative of antibody aggregates (Fig. 2c). Therefore, HLA-A3 single-positive fluorescent events were interpreted to not be representative of EVs, and were excluded from further analysis.

Analysis of CD9 + HLA-A3 + double-positive fluorescent event concentrations in the KTR group showed that our assay produced background signals that varied among the different recipients (recipient-specific background) despite recipients all being HLA-A3 negative. Luminex single antigen assay for the anti-HLA-A3 antibody showed no cross reactivity of the antibody with other HLA epitopes (Supplementary Data S1 online), thus validating the specificity of the antibody. Comparison of CD9 + HLA-A3 + concentrations between the donor and recipient groups (1.60E^7^ ± 1.03E^7^ objects/mL vs 4.29E^6^ ± 4.48E^6^ objects/mL, respectively) showed that these double-positive fluorescent events can be used to discriminate samples on the basis of HLA phenotype. Concentrations of CD9 + HLA-A3 + double-positive events as measured in donor PPP samples showed an approximately 96% reduction after detergent treatment, with concentrations after detergent treatment residing in the range of the isotype and buffer controls (Fig. 2d). Consequently, CD9 + HLA-A3 + double-positive fluorescent events were identified to represent EVs.

It should be noted that some samples showed low/minimal reduction in concentrations of fluorescent events detected after detergent treatment (as illustrated by 1 recipient sample in Fig. 2d). These events were interpreted to not represent membrane-delimited vesicular events, but rather be indicative for protein complexes (which are unaffected by detergent treatment). To identify and exclude such samples from future analysis, we established a threshold of ≥ 95% reduction (in concentration) after detergent treatment for samples to be included in future analysis. This cut-off value was established to ensure that only samples are included that are representative (≥ 95%) of membrane-delimited particles (as opposed to protein complexes).

These observations show that our IFCM protocol is able to identify and discriminate single EVs in diluted PPP samples on the basis of their HLA phenotype by analyzing events that are both CD9 + (plasma EV marker) and HLA-A3 + (to discriminate between HLA-A3 + and HLA-A3 −  individuals).

### IFCM discriminates single CD9 + HLA-A3 + EVs down to 1% above recipient-specific background

To determine the discriminative power of our IFCM protocol to detect CD9 + HLA-A3 + EVs above (recipient-self) background signals in KTR samples, we serially diluted (twofold dilutions) HLA-A3 + PPP into HLA-A3 − PPP (5 matched donor-recipient couples), labelled these samples with anti-CD9 and anti-HLA-A3, and analyzed these with IFCM.

**Figure 3:**
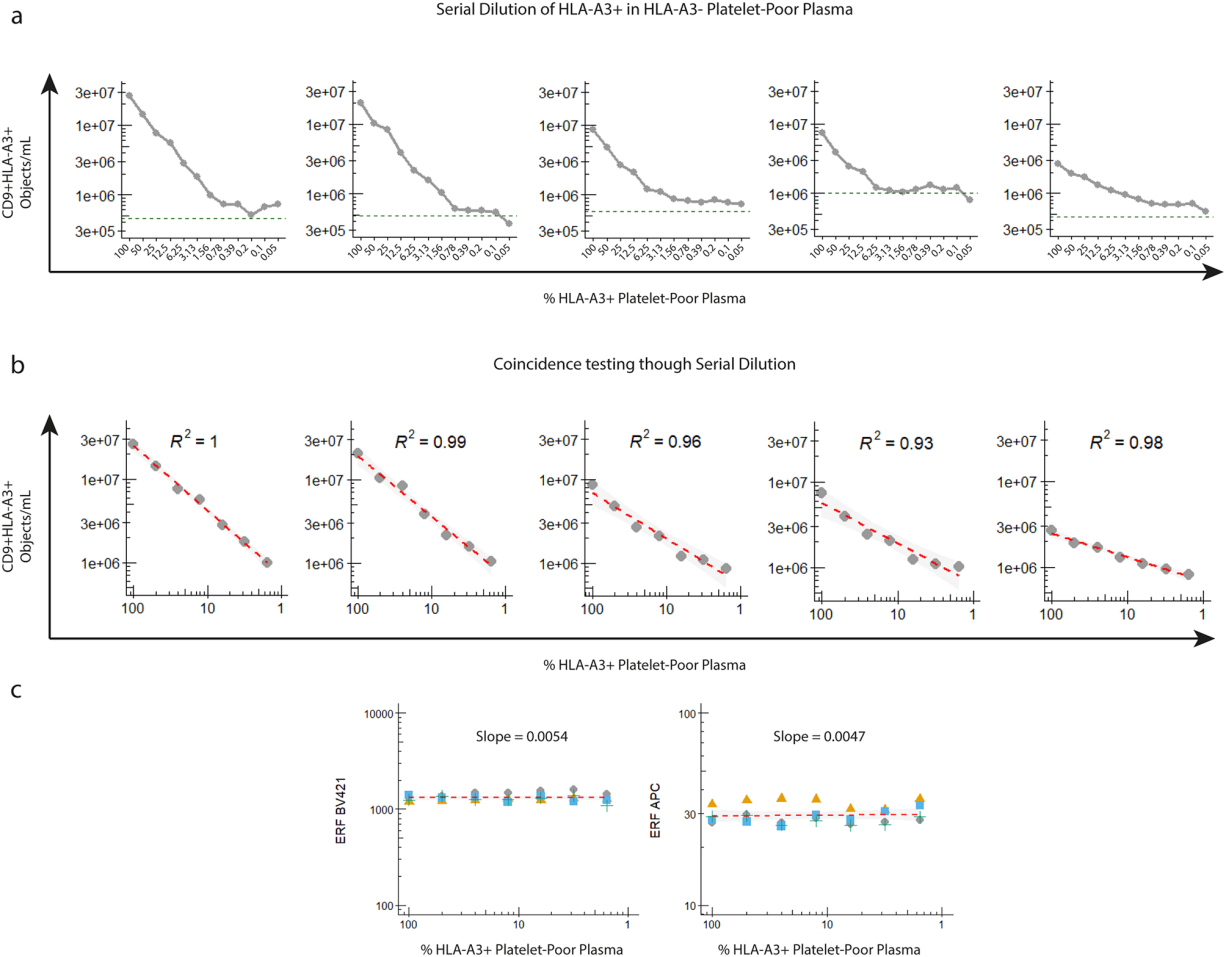
Quantification of CD9 + HLA-A3 + events in mixed PPP samples. (**a**) Serial dilutions (twofold) of 5 double-stained (anti-CD9 and anti-HLA-A3) matched donor-recipient samples shows that our setup is able to discriminate CD9 + HLA-A3 + EVs above recipient ‘self’ background (HLA-A3- recipient signals, indicated by the green dashed lines) down to ~ 1%. (**b**) Coincidence testing through serial dilution. A linear correlation between the concentrations of CD9 + HLA-A3 + EVs and dilution factor (range: 100% to 1.56% HLA-A3 + PPP) for each serial dilution experiment confirmed single particle analysis in mixed PPP samples. (**c**) Stable (standardized) fluorescent signals for both detection channels (Ch01 – HLA-A3 / Ch05 – CD9) were observed irrespective of the dilution factor.

For each of the 5 donor-recipient couples, we quantified the concentrations of CD9 + HLA-A3 + EVs as detected for each dilution step (Fig. 3a). Similar to the previous observation, we observed that each of the 5 HLA-A3 − PPP recipient samples (without any spiked-in HLA-A3 + PPP) yielded varying background concentrations. These were interpreted to represent the recipient-self sample-specific background (indicated with dashed green lines, Fig. 3a). We were able to detect CD9 + HLA-A3 + EVs above their respective sample-specific background concentrations down to ~ 100-fold dilution, thus with CD9 + HLA-A3 + EVs being detectable ~ 1% above the recipient ‘self’ signal.

Next, we examined the accuracy of our IFCM protocol to detect single CD9 + HLA-A3 + EVs in mixed plasma samples – as opposed to the coincidence detection of multiple particles recorded as a single event. For each individual (donor-in-recipient) dilution experiment, the concentrations of CD9 + HLA-A3 + EVs as detected for each dilution step (range: 100–1.56% HLA-A3 + PPP) were analyzed using a linear regression model (Fig. 3b). We observed that the concentrations of CD9 + HLA-A3 + EVs were linearly proportional to the dilution factor, as indicated by the R^2^ values. Additionally, analysis of the (standardized) fluorescent intensities as detected in both detection channels revealed that the fluorescent intensities were unaffected by serial dilution; mean: 1315 (range 1085–1604) ERF for BV421 (HLA-A3), and 29.5 (range 25.7–35.8) ERF for APC (CD9) (Fig. 3c).

Taken together, these data indicate that IFCM is able to detect single dd-EVs directly in recipients’ circulation if their fraction exceeds ~ 1% above their recipient specific background.

### dd-EVs are detected in KTRs with stable allograft function

Next, we examined whether dd-EVs could be directly detected in PPP of KTRs after transplantation. As stated previously, samples not passing the threshold of ≥ 95% reduction after detergent treatment were excluded from analysis; excluded samples were not associated with any specific time-point. Sample exclusion on the basis of detergent treatment resulted in the exclusion of 10 KTR sample series, leaving 26 KTR sample series in the analysis. Table 1 shows the patient characteristics corresponding to the samples included in our analysis, stratified into the four groups as described in the methods section.

**Table 1:** Patient characteristics corresponding to the samples included in our analysis after exclusion of samples not passing the 95% reduction after detergent treatment.

	Control	Other diagnosis	Presumed rejection	Rejection	*p-*test
n =	11	3	5	7	
Recipient age (Years)	58.64 (10.06)	62.33 (10.21)	61.20 (14.55)	58.29 (16.43)	0.95
Recipient gender (Male)	9 (81.8)	3 (100.0)	1 (20.0)	3 (42.9)	0.04
Recipient BMI	27.45 (5.13)	28.03 (11.94)	26.62 (2.27)	27.44 (4.92)	0.99
**Donor type**					0.60
DBD	3 (27.3)	1 (33.3)	0 (0.0)	3 (42.9)	
DCD	3 (27.3)	2 (66.7)	1 (20.0)	2 (28.6)	
Living donor-related	1 (9.1)	0 (0.0)	1 (20.0)	0 (0.0)	
Living donor-unrelated	4 (36.4)	0 (0.0)	3 (60.0)	2 (28.6)	
Donor age (Years)	54.09 (17.68)	66.00 (2.65)	61.20 (13.55)	64.00 (6.90)	0.38
Donor gender (Male)	4 (36.4)	1 (33.3)	4 (80.0)	3 (42.9)	0.40
**Missmatch HLA-A**					0.217
0	0 (0.0)	0 (0.0)	1 (20.0)	0 (0.0)	
1	9 (81.8)	2 (66.7)	1 (20.0)	4 (57.1)	
2	2 (18.2)	1 (33.3)	3 (60.0)	3 (42.9)	
**Missmatch HLA-B**					0.56
0	2 (18.2)	0 (0.0)	0 (0.0)	0 (0.0)	
1	4 (36.4)	1 (33.3)	1 (20.0)	4 (57.1)	
2	5 (45.5)	2 (66.7)	4 (80.0)	3 (42.9)	
**Missmatch HLA-DR**					0.02
0	4 (36.4)	0 (0.0)	0 (0.0)	3 (42.9)	
1	5 (45.5)	0 (0.0)	5 (100.0)	2 (28.6)	
2	2 (18.2)	3 (100.0)	0 (0.0)	2 (28.6)	
**Induction therapy**					
Basiliximab	11 (100.0)	3 (100.0)	5 (100.0)	5 (71.4)	0.12
Alemtuzumab	0 (0.0)	0 (0.0)	0 (0.0)	2 (28.6)	0.12
**Maintenance immunosuppression**					
TAC / MMF / Prednisolone	11 (100.0)	3 (100.0)	5 (100.0)	7 (100.0)	NA

**Figure 4:**
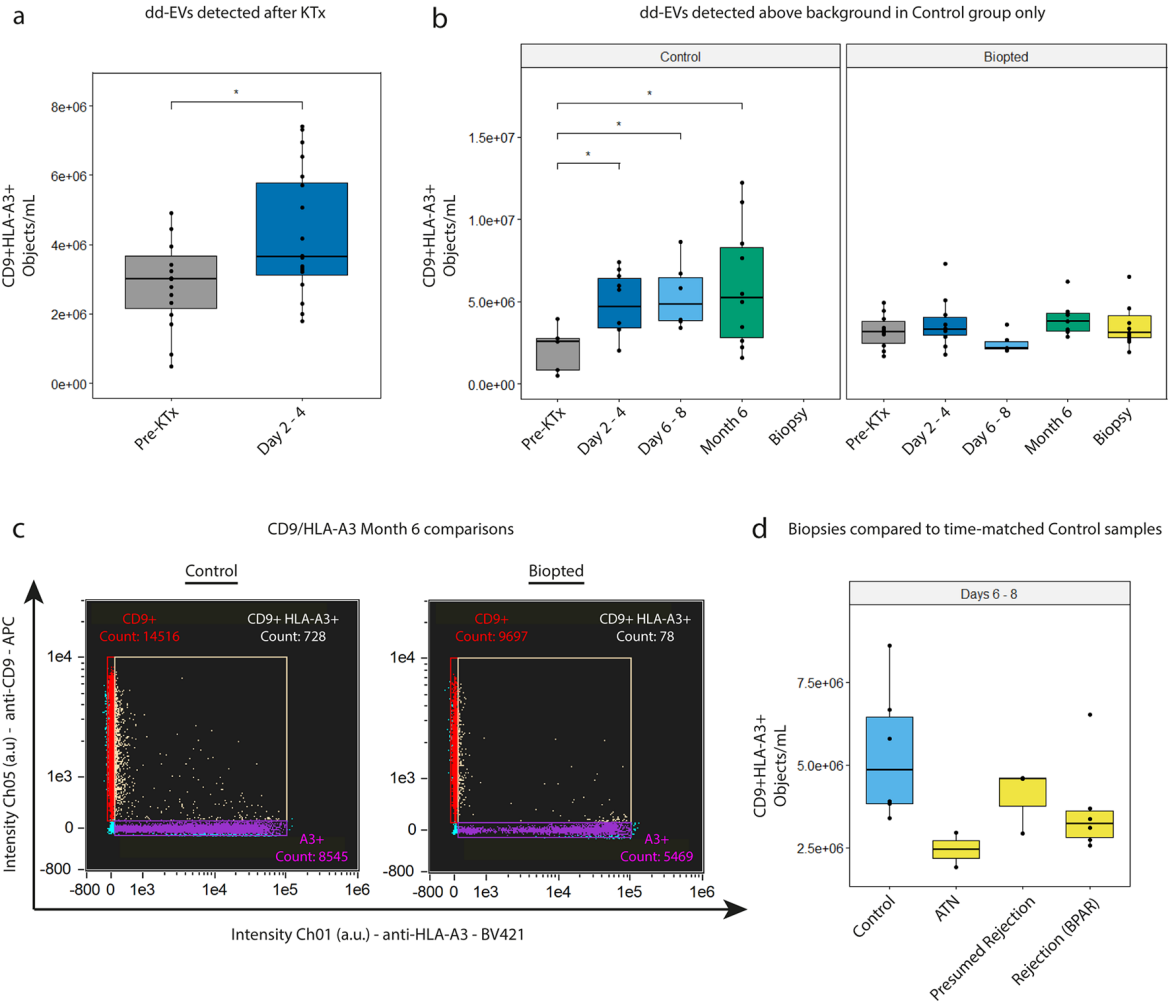
Detection of dd-EVs after transplantation. (**a**) Analysis of the concentrations of CD9 + HLA-A3 + events before and 2–4 days after kidney transplantation revealed a significant increase after transplantation. (**b**) longitudinal analysis of dd-EV concentrations in KTR blood samples subdivided into either (1) the control group (representing all samples from patients whom did not experience any complications after transplantation) or (2) the biopted group (encompassing all samples from patients whom underwent a biopsy), showing that dd-EVs are detectable above baseline (before transplantation) only in patients whom did not experience complications after transplantation. (**c**) Representative CD9 vs HLA-A3 scatter plots of PPP samples taken 6 months after KTx from an individual in the control (left) and biopted (right) group. (**d**) dd-EV concentrations as detected in ‘for-cause’ biopsy samples compared to time-matched control samples.

For the dd-EV analysis, samples from a total of 26 KTRs were included in the analysis (Fig. 4B). Continued variables are described as mean (SD). Categorical variables as number of cases (%).

*ATG* Antithymocyte globulin, *BMI* Body mass index, *DBD* Donation after brain death, *DCD* Donation after circulatory death, *MMF* Mycophenolate mofetil, *MPA* Mycophenolic Acid, *TAC* Tacrolimus.

First, we compared the total concentrations of double-positive events measured in PPP samples taken before (n = 13) and 2–4 days (n = 17) after transplantation (Fig. 4a). We observed a statistically significant difference between these time points which confirms the release of dd-EVs into KTR's circulation.

We then performed a longitudinal analysis of dd-EV concentrations comparing KTRs in the control group with KTRs who underwent a ‘for-cause’ kidney biopsy (‘Biopted’ group) (Fig. 4b). In the control group, we observed significant increases in dd-EV concentrations at 2–4 days, 6–8 days and 6 months after transplantation compared to the concentrations detected before transplantation. These dd-EV concentrations were observed to be stable throughout follow-up, suggesting that stable allograft function (without a biopsy) leads to a detectable dd-EV signal in KTR plasma. This notion was further strengthened by the observation that dd-EV concentrations did not increase (compared to levels detected before transplantation) in individuals who did experience allograft complications (‘Biopted’ group); this effect was observed up to at least 6 months after transplantation (Fig. 4c).

To examine a potential diagnostic value of dd-EVs, we next compared the concentrations of CD9 + HLA-A3 + EVs measured in ‘for-cause’ biopsy samples taken at 6 days after transplantation (median, range: 2–60) with the concentrations as detected in samples taken 6–8 days after transplantation from patients in the ‘control’ group.

The concentration of dd-EVs was higher in time-matched KTR samples without a biopsy compared to concentrations detected at the moment of a for-cause biopsy (irrespective of their pathological classification, 5.36E^6^ ± 2.05E^6^ objects/mL vs 3.24E^6^ ± 8.55E^5^ objects/mL, respectively, *p *= 0.05). However, no statistical differences were observed when comparing dd-EV concentrations between the control samples and biopsies indicative for ATN (N = 2), Presumed Rejection (N = 3), or Biopsy Proven Acute Rejection (BPAR—3 × aABMR and 3 × aTCMR2a) , demonstrating that dd-EVs were unable to discriminate between the type of complication (Fig. 4d).

## Discussion

EVs have great potential value as (minimally invasive) biomarkers^26^, but sensitive and reproducible methods for single EV analysis are essential to understand the role of EVs in human health and disease^22^. In the current work, we assessed the applicability of our recently developed IFCM-based methodology^23^ to directly measure EV subsets in human patient plasma samples – as exemplified by the detection of single dd-EVs in KTR plasma samples after kidney transplantation.

In addition to their physical properties and current technological challenges, the detection of EV subsets during health and disease may – depending on the disease – be hampered by the presence of ‘contaminating’ agents in the sample matrix. In the current study, components such as urea and creatinine are elevated as a consequence of kidney failure and may interfere with single EV detection. We show that our IFCM protocol is able to detect single CD9 + EVs in plasma samples obtained from both donors (healthy controls) and KTRs before transplantation, thereby suggesting that single EV detection by our IFCM protocol is uninfluenced by such contaminating components. The concentration of EVs in urine is related to nephron mass, explaining a lower concentration of total CD9 + EVs in KTRs compared to donors^27^. Here, we did not observe a difference in total CD9 + EVs between both groups in blood plasma, which is most likely due to the contribution of multiple organs to the total plasma CD9 + EV pool.

The concept of plasma circulating dd-EV detection and characterization with IFCM has been presented previously^4^. Mastoridis et al. showed that by analyzing CFSE + events in combination with an exosome-specific marker (CD63) and an origin-specific marker (donor-HLA), circulating dd-EVs could be detected in the circulation of a liver transplantation recipient^4^. However, our protocol provides several improvements over the approach presented in previous work: (1) our IFCM platform is both SSC (size) and fluorescence calibrated—which enhances reproducibility, (2) no EV isolation was performed—thus the ‘full spectrum’ of detectable EVs was analyzed, and (3) by analyzing CD9 + EVs (shown to be highly prevalent in plasma samples)^4,23^ in combination with donor-HLA, we were able to analyze a broad spectrum of circulating dd-EVs – as opposed to the analysis of donor-derived CFSE + CD63 + exosomes (a donor EV subset). Additionally, we prove the detection of single dd-EVs by our IFCM protocol and determined its sensitivity through serial dilution of HLA-A3 + plasma into HLA-A3 −  plasma demonstrating a linear correlation with the dilution factor and stable ERF^22^.

Currently, it is consensus that CD9 + EVs detected in PPP are most likely platelet-derived. As platelets express HLA Class I antigens on their surface, one might expect a high degree of co-localization of HLA Class I antigens with CD9 on these EVs. However, we found that CD9 + HLA-A3 + particles are only a fraction (~ 16%) of the total CD9 + particles in HLA-A3 + donors. This observation may be explained by (1) different modes of EV biogenesis influencing whether HLA Class I antigens are present on EVs and/or whether their epitope topology is directed to the vesicular surface (and thus detectable with our setup), and (2) CD9 (but not HLA Class I antigens) may also be found on lipoproteins^18^.

The identification and quantification of single dd-EVs with IFCM as presented here is also subject to limitations. First, IFCM needs a minimum of 3 pixels before an event is recorded^23^. Consequently, EVs with a low HLA-A3 epitope-density might be missed by our assay. Second, although we calibrated the arbitrary fluorescent SSC intensities of our platform to reflect particle-size, we obtained a goodness-of-fit measure (R^2^) of 91%, which implies a 9% error when selecting particles with SSC intensities corresponding to EVs ≤ 400 nm^23^. For reference, (conventional) clinical-grade Flow Cytometers typically result in R^2^ values > 0.99—although lacking the resolution to detect EVs < 300 nm^18,28,29^. Additionally, we excluded samples not passing the threshold of ≥ 95% reduction after detergent treatment from analysis, which imposes an analytical bias towards samples containing ‘high’ concentrations of fluorescent events. However, by applying this selection criteria on our samples we ensured the analysis of fluorescent events of biological origin well above the (fluorescent) background of our assay. An alternative to the detergent treatment threshold could be the selective analysis of samples with a minimum number of events in the gate of interest (exceeding the number of events acquired in negative control samples e.g., buffer + reagents, unstained plasma, plasma + isotype staining) after acquisition.

In transplantation, the potential of EVs as biomarker for the detection of allograft rejection has been reported by a few groups^30–36^. Most notably, animal models of heterotopic heart transplantation (mouse into mouse)^24^, islet xenotransplantation (human into mouse)^14^, and a lung transplantation model between rats (Wistar into Lewis)^25^, have provided evidence that concentrations of allograft-derived EVs diminish during rejection well before alterations in classical biomarkers occur or histologic manifestations of injury were observed. In the current study, we observed a stable release of dd-EVs in KTRs who did not experience allograft dysfunction after transplantation but were unable to detect dd-EVs above pre-transplantation signals in KTRs who underwent a ‘for-cause’ biopsy.

Mechanistically, it is currently unknown whether the lower concentrations of dd-EVs during allograft dysfunction is a consequence of decreased production of EVs by the allograft, increased consumption of dd-EVs by recipient immune cells, or a combination of both^24^. Donor exosomes have been shown to be involved in donor antigen presentation to recipient alloreactive T cells in lymphoid organs by the recipient dendritic cells in a phenomenon known as cross-dressing^35,37–39^. As Habertheuer et al. suggested, this mechanism of T cell activation may suppress production of exosomes by the transplanted tissue even before there is targeted injury to the allograft^24^. At any rate, the detection of dd-EVs can be associated with stable allograft function – which is in contrast to the detection of e.g. donor-derived cell-free DNA, which has been observed to increase in concentration as a consequence of allograft damage^40^.

In conclusion, our calibrated IFCM-based methodology to directly detect and characterize plasma-derived EV subsets is applicable in patient human plasma samples. We believe that this methodology—after validation of markers of interest—could boost the EV biomarker research in a variety of clinical contexts, of which monitoring of kidney transplant integrity appears especially promising.

## Materials and methods

### Clinical sample selection

To analyze distinct donor-derived EVs (dd-EVs) in KTR plasma samples, 36 donor-KTR couples were selected on the basis of an HLA-A3 mismatch between donors (HLA-A3 +) and KTRs (HLA-A3 − ). KTRs had not received a previous HLA-A3 + graft. All donor-KTR couples participated in an observational study which aimed to identify minimally invasive biomarkers for the diagnosis of acute kidney transplant rejection, and was approved by the institutional review board of the Erasmus MC (Medical Ethical Review Board number 2018-035); details of this study are described elsewhere^40^. All patients provided written informed consent. Blood samples from living donors were obtained before donation and collected as part of our ongoing Biobank program (Medical Ethical Review Board number 2010-022). These donors served as healthy controls. The study was conducted in accordance with the principles of the Declaration of Helsinki.

### Sample collection and processing

From both donors and KTRs, whole blood samples (EDTA) were collected before transplantation, and, for KTRs, 3 days, 7 days, and 6 months after transplantation. Additionally, blood samples were collected on the morning of (or the day preceding) a ‘for-cause’ kidney transplant biopsy.

Blood was drawn from each individual into two BD Vacutainer K3-EDTA-coated collection tubes (BD Biosciences, San Jose, USA). Whole blood was centrifuged at 1910 × *g* for 10 min at room temperature and the plasma layer was then collected at 16,000 × *g* for 10 min at room temperature in 1 mL aliquots using Safe-Lock Eppendorf tubes (Eppendorf AG, Hamburg, Germany). The resulting platelet-poor plasma (PPP) was first pooled before being divided into 700-µL aliquots in cryovials containing 28 µL of a 25 × concentrated protease inhibitor cocktail solution (4% v/v) (cOmplete Protease inhibitor cocktail tablets, Roche, Mannheim, Germany) according to the manufacturers’ instructions and stored at − 80 °C.

### Stratification of KTRs based on biopsy scores

Biopsies were scored by an experienced renal pathologist, and KTRs were divided into 4 groups based on (1) no ‘for-cause’ biopsy and no anti-rejection therapy (‘Control’ group), (2) acute tubular necrosis (ATN) as the main finding in the biopsy and no anti-rejection therapy (‘ATN’ group), (3) no histopathological signs of rejection but treated with anti-rejection therapy on the basis of clinical suspicion of rejection (‘Presumed Rejection’ group), and (4) biopsy-proven acute rejection (BPAR—aABMR or aTCMR2A) in combination with anti-rejection therapy (‘Rejection’ group). In case patients underwent multiple biopsies over the course of the study, only the first biopsy was used in the analysis.

### Sample labelling

PPP samples were stained with monoclonal antibodies (mAbs) and isotype controls as extensively described in our previous work^23^. In the absence of a specific marker, EVs are identified by their expression of common markers such as CD9, CD63 and CD81^41^. In this study, we used CD9 (which has been shown to be highly prevalent on plasma-derived EVs) as common EV marker^23^. Additionally, following the characterization of kidney-derived EVs released during normothermic machine perfusion, we determined that CD9 co-localizes to a higher degree with MHC Class I molecules compared to CD63 (~ threefold difference) (Supplementary Figure S1online). mAbs used in this study were CD9–APC, clone HI9a (6 µg/mL, Biolegend, San Diego, USA), and HLA-A3-BV421, clone GAP-A3 (200 µg/mL, BD Biosciences, New York, USA). Matched isotype controls were IgG1, k-APC, clone MOPC-21 (200 µg/mL, BioLegend), and IgG2a, k-BV421, clone G155-178 (200 µg/mL, BD Biosciences). Specificity of the anti-HLA-A3 mAbs was confirmed by Luminex single antigen assay (Supplementary Data S1 online). Prior to staining, mAbs and isotypes were centrifuged at 16,000 × *g* for 10 min at room temperature to remove potential mAb clumps (to reduce false-positive signals from analysis), and were diluted in 0.20 µm filtered PBS (fPBS) before staining (final concentrations: 200 ng/mL).

Staining was performed by addition of the diluted mAbs/isotypes to 30 µL of PPP followed by a pre-determined volume of fPBS (V_tot_ = fPBS + sample + mAbs = 130 µL) followed by O/N incubation at 4 °C. All samples were brought to a total volume of 380 µL using fPBS before IFCM measurements.

### Controls

To ascertain EV measurements the following controls were applied, as recommended by the MIFlowCyt-EV framework^22^: buffer only, buffer with reagents, unstained samples, isotype controls, and detergent treatment, which aims to disrupt the membranous structure of EVs thereby allowing discrimination between membrane-enclosed vesicles (which lyse upon detergent treatment) and other protein complexes (which are unaffected by detergent treatment). Detergent treatment was performed by adding 20 µL of 10% (V/V) TritonX-100 to the samples followed by 30 min of incubation at room temperature prior to acquisition.

### Data acquisition

All samples were acquired on an ImageStreamX MkII instrument (IS^x^; Luminex). Settings as extensively described elsewhere were used^23^. In brief, lasers were turned on as applicable per fluorophore and set to their maximum power (405 nm : 200 mW, 642 nm :150 mW) with the exception of the 785 nm SSC laser (1.25 mW). High Gain mode—an upgrade of the IFCM that increases the photonic sensitivity and object detection of the system – was activated. Data was acquired over fixed time periods – to standardize among samples – of 180 s using the 60 × objective with fluidics set to ‘low speed / high sensitivity’. This resulted in a flow speed of 43.59 ± 0.07 mm/sec (mean ± standard deviation). Core size was set at 6 µm, autofocus was activated and the ‘Remove Speedbead’ option was checked. BV421 fluorescence signals were collected in channel 1 (435–505-nm filter), APC signals in channel 5 (642–745 nm filter), and SSC signals in channel 6 (745–785 nm filter). Particle enumeration was achieved through the advanced fluidic control of the IS^x^ coupled with continuously running speed beads, resulting in the “objects/mL” feature within the IS^x^ Data Exploration and Analysis Software (IDEAS).

## Data analysis

Data analysis was performed using Amnis IDEAS software (version 6.2). Image display mapping was linearly adjusted for all fluorescent events for each channel and then applied to all files of the respective experiment. To ensure the analysis of EVs we (1) selected all particles with SSC intensities ≤ 900 a.u., and (2) identified and excluded coincidence detection by counting the number of fluorescent spots within the pixel grid for each event acquired; events showing multiple spots were excluded from analysis^23^. This gating strategy ensures the selection and analysis of single spot fluorescent particles ≤ 400 nm. Gating areas and cut-offs were established through identification of (fluorescent) populations in unstained and single stained samples, and arbitrary fluorescent intensities were converted into Equivalent number of Reference Fluorophores (ERF) values based on previously published calibration data^23^. Lower and upper gating area cut-offs were defined as 677 – 112,201 ERF for BV421, and 6.40 – 123 ERF for APC.

### Statistical analysis

Statistical analysis was performed using R version 4.0.2 and RStudio (RStudio Team (2016). RStudio: Integrated Development for R. RStudio, Inc., Boston, MA URL http://www.rstudio.com/.) version 1.1.463. All concentrations reported in this work were corrected for sample dilution (before acquisition − 380 µL total volume per test containing 30 µL sample =  ~ 12.33-fold dilution factor) and are shown as the mean ± standard deviation unless specified otherwise. Statistical significance between EV concentrations and groups was determined through two-sided t-tests, 95% CI with unpaired data.

## Supplementary Information

Supplementary Information 1.

Supplementary Information 2.

## Data Availability

All source data underlying the figures presented in this work are provided as ‘Supplementary Data Figs. 1–4’. Any other relevant data are available from the corresponding author upon reasonable request.

## Abbreviations

EVs: Extracellular vesicles
dd-EVs: donor-derived extracellular vesicles
IFCM: Imaging flow cytometry
KTR: Kidney transplant recipient
HLA: Human leukocyte antigen
PPP: Platelet-poor plasma
SSC: Side scatter
