# Supplementary material for: Direct detection of circulating donor-derived extracellular vesicles in kidney transplant recipients
Source: Sci Rep. 2022 Dec 20;12:21973. doi: 10.1038/s41598-022-26580-6 (PMC9768203; doi:10.1038/s41598-022-26580-6)
Supplement: Supplementary file 2 — Supplementary Information 2. [file 41598_2022_26580_MOESM2_ESM.pdf]

DIRECT DETECTION OF DONOR-DERIVED EXTRACELLULAR VESICLES IN KIDNEY TRANSPLANTATION: TOWARDS CLINICAL APPLICABILITY

WOUTER W. WOUD, MSC[1*], DENNIS A. HESSELINK, PHD, MD[1], MARTIN J. HOOGDUIJN, PHD[1], CARLA C. BAAN, PHD[1], KARIN BOER, PHD[1]

[1]Erasmus MC Transplant Institute, Department of Internal Medicine, University Medical Center Rotterdam, Rotterdam, The Netherlands

SUPPLEMENTARY FILES

Supplementary Data S1

LUMC

'

Class I Single Antigen Results

Batch ID: L1_LC_24-08-2021_I

Patient Name: A3-PE 150 **Patient DOB:** **Draw Date:** **Donor Number:** **Expiration Date:** 14-01-2022

Sample ID: A3-PE 150 **Lot ID:** 3010654 3010500-SA1 **Donor Center # :** **Accession # :**

Patient HLA Type: **Report By:** Lab Supervisor

Method Type: Standard Calculation

Positive CON MFI : 1 **Negative CON MFI :** 0 **Analysis Mode:** Manual **% PRA:** 1

Bead Count	Antigen ID	Cut-off	Raw Value	MFI/LRA	Assignment	BG Adjusted	AD-MFI	AD-BG Adjusted	A	B	C	Bw	A Serology	B Serology	C Serology	Epitopes
72	109	3.49	8722	8722.00	Positive	8554	7565	7419	*A*03:01*				A3			161D
82	191	3.61	11	11.00	Negative	-276	20	-495.51			C*07:02				Cw7	
97	181	4.04	10	10.00	Negative	-187	9	-171.72		B*82:02		Bw6				
82	170	3.98	9	9.00	Negative	-201	9	-197.64		B*53:01		Bw4		B53		
78	190	3.65	8	7.50	Negative	-288	12	-472.09			C*07:01				Cw7	
80	180	3.73	7	7.00	Negative	-174	6	-160.81		B*81:01		Bw6		B81		
81	198	3.95	7	7.00	Negative	-350	12	-602.41			C*17:01					
76	199	3.45	7	7.00	Negative	-254	9	-329.44			C*18:01					
86	159	4.30	6	6.00	Negative	-234	6	-231.45		B*42:01		Bw6		B42		
104	169	4.34	6	6.00	Negative	-222	6	-212.85		B*52:01		Bw4		B52(5)		
96	179	3.90	5	5.00	Negative	-199	5	-193.02		B*78:01		Bw6		B78		
60	197	3.41	5	5.00	Negative	-245	7	-361.36			C*16:01					
80	149	3.83	4	4.00	Negative	-192	4	-172.20		B*27:05		Bw4		B27		
95	168	4.26	4	4.00	Negative	-210	4	-205.68		B*51:01		Bw4		B51(5)		
83	189	3.34	4	4.00	Negative	-268	4	-291.94			C*06:02				Cw6	
78	196	3.35	4	4.00	Negative	-242	5	-292.62			C*15:02					
83	148	3.84	3	3.00	Negative	-184	2	-152.82		B*27:03		Bw4		B27		
94	157	4.04	3	3.00	Negative	-204	3	-185.29		B*40:02		Bw6		B61(40)		
82	158	4.31	3	3.00	Negative	-231	3	-250.81		B*41:01		Bw6		B41		
83	167	3.99	3	3.00	Negative	-193	3	-166.24		B*50:01		Bw6		B50(21)		
98	177	3.40	3	3.00	Negative	-153	2	-123.19		B*67:01		Bw6		B67		
62	178	4.21	3	3.00	Negative	-209	3	-196.61		B*73:01				B73		
97	185	3.47	3	3.00	Negative	-260	3	-269.99			C*03:04				Cw10(w3	
95	188	3.64	3	3.00	Negative	-289	5	-493.17			C*05:01				Cw5	
78	195	3.39	3	3.00	Negative	-252	3	-278.15			C*14:02					
96	176	3.73	3	2.50	Negative	-183	2	-165.76		B*59:01		Bw4		B59		
103	138	3.99	2	2.00	Negative	-187	2	-158.88		B*14:01		Bw6		B64(14)		
79	139	4.73	2	2.00	Negative	-262	2	-260.44		B*14:02		Bw6		B65(14)		
77	147	3.90	2	2.00	Negative	-195	2	-170.45		B*18:01		Bw6		B18		
84	156	3.69	2	2.00	Negative	-176	2	-150.04		B*40:01		Bw6		B60(40)		
93	161	4.27	2	2.00	Negative	-228	2	-193.71		B*44:03		Bw4		B44(12)		
81	164	3.93	2	2.00	Negative	-201	2	-185.08		B*47:01		Bw4		B47		
98	165	4.38	2	2.00	Negative	-270	3	-355.26		B*48:01		Bw6		B48		
104	166	3.80	2	2.00	Negative	-179	2	-148.55		B*49:01		Bw4		B49(21)		
106	173	4.00	2	2.00	Negative	-194	2	-181.99		B*56:01		Bw6		B56(22)		
94	174	3.76	2	2.00	Negative	-186	2	-160.62		B*57:01		Bw4		B57(17)		
81	175	4.06	2	2.00	Negative	-204	2	-189.59		B*58:01		Bw4		B58(17)		
76	182	3.41	2	2.00	Negative	-266	2	-301.59			C*01:02				Cw1	
83	184	3.44	2	2.00	Negative	-252	2	-290.32			C*03:03				Cw9(w3)	
102	186	3.86	2	2.00	Negative	-330	6	-988.02			C*04:01				Cw4	
70	187	3.42	2	2.00	Negative	-259	3	-331.63			C*04:03					
86	192	3.29	2	2.00	Negative	-236	2	-290.64			C*08:01				Cw8	
86	194	3.51	2	2.00	Negative	-252	2	-268.09			C*12:02					
100	117	3.94	2	1.50	Negative	-212	2	-241.16	A*29:01				A29(19)			
76	183	3.55	2	1.50	Negative	-269	3	-500.00			C*02:02				Cw2	
72	108	4.03	1	1.00	Negative	-215	1	-197.43	A*02:05				A2			
100	110	3.64	1	1.00	Negative	-169	1	-146.07	A*11:01				A11			
81	111	3.77	1	1.00	Negative	-195	1	-180.56	A*11:02				A11			
73	114	3.74	1	1.00	Negative	-190	1	-166.96	A*24:03			Bw4	A2403			
81	115	3.47	1	1.00	Negative	-172	1	-157.08	A*25:01			Bw4	A25(10)			
82	116	3.65	1	1.00	Negative	-187	1	-178.61	A*26:01				A26(10)			
93	118	4.07	1	1.00	Negative	-220	1	-213.59	A*29:02				A29(19)			
78	119	4.02	1	1.00	Negative	-213	1	-217.57	A*30:01				A30(19)			
90	120	4.31	1	1.00	Negative	-234	1	-237.08	A*31:01				A31(19)			
88	121	3.73	1	1.00	Negative	-186	1	-190.96	A*32:01			Bw4	A32(19)			
73	122	3.89	1	1.00	Negative	-204	1	-221.26	A*33:01				A33(19)			
80	123	4.23	1	1.00	Negative	-231	1	-230.77	A*33:03				A33(19)			

88	124	3.82	1	1.00	Negative	-194	1	-199.79	A*34:02				A34(10)			
97	125	4.06	1	1.00	Negative	-215	1	-202.64	A*36:01				A36			
85	126	4.25	1	1.00	Negative	-217	1	-217.00	A*43:01				A43			
82	128	3.60	1	1.00	Negative	-167	1	-143.47	A*66:02				A66(10)			
100	129	3.70	1	1.00	Negative	-190	1	-192.31	A*68:01				A68(28)			
94	130	3.51	1	1.00	Negative	-168	1	-152.17	A*68:02				A68(28)			
90	134	4.61	1	1.00	Negative	-253	1	-233.61		B*07:02		Bw6		B7		
90	135	4.09	1	1.00	Negative	-196	1	-170.88		B*07:03		Bw6		B703		
88	136	3.71	1	1.00	Negative	-177	1	-144.61		B*08:01		Bw6		B8		
75	137	4.25	1	1.00	Negative	-214	1	-219.26		B*13:02		Bw4		B13		
92	140	3.59	1	1.00	Negative	-171	1	-138.46		B*15:01		Bw6		B62(15)		
68	142	3.82	1	1.00	Negative	-186	1	-149.88		B*15:03		Bw6		B72(70)		
94	143	3.95	1	1.00	Negative	-189	1	-149.76		B*15:12		Bw6		B76(15)		
72	144	4.17	1	1.00	Negative	-212	1	-200.19		B*15:13		Bw4		B77(15)		
65	145	4.32	1	1.00	Negative	-205	1	-169.28		B*15:16		Bw4		B63(15)		
92	146	3.92	1	1.00	Negative	-184	1	-148.51		B*15:18		Bw6		B71(70)		
92	150	3.56	1	1.00	Negative	-149	1	-107.27		B*27:08		Bw6		B2708		
79	151	4.17	1	1.00	Negative	-227	1	-228.83		B*35:01		Bw6		B35		
83	152	4.28	1	1.00	Negative	-233	1	-256.33		B*35:08		Bw6		B35		
78	153	3.78	1	1.00	Negative	-186	1	-161.46		B*37:01		Bw4		B37		
83	155	3.66	1	1.00	Negative	-167	1	-136.44		B*39:01		Bw6		B3901		
82	160	4.07	1	1.00	Negative	-208	1	-190.48		B*44:02		Bw4		B44(12)		
98	162	4.17	1	1.00	Negative	-214	1	-231.60		B*45:01		Bw6		B45(12)		
82	163	5.05	1	1.00	Negative	-303	1	-388.96		B*46:01				B46		
87	171	3.91	1	1.00	Negative	-194	1	-175.25		B*54:01		Bw6		B54(22)		
74	172	4.29	1	1.00	Negative	-239	1	-258.10		B*55:01		Bw6		B55(22)		
81	193	3.52	1	1.00	Negative	-268	1	-342.71			C*08:02				Cw8	
90	103	4.00	1	0.50	Negative	-217	0	-209.38	A*01:01				A1			
93	104	3.82	0	0.00	Negative	-204	0	-219.12	A*02:01				A2			
100	106	3.89	0	0.00	Negative	-204	0	-185.45	A*02:02				A2			
101	107	3.64	0	0.00	Negative	-198	0	-182.49	A*02:03				A203			
111	112	3.52	0	0.00	Negative	-174	0	-177.37	A*23:01			Bw4	A23(9)			
106	113	3.83	0	0.00	Negative	-189	0	-179.49	A*24:02			Bw4	A24(9)			
71	127	3.56	0	0.00	Negative	-168	0	-144.70	A*66:01				A66(10)			
106	131	4.19	0	0.00	Negative	-229	0	-275.57	A*69:01				A69(28)			
108	132	3.77	0	0.00	Negative	-191	0	-194.90	A*74:01				A74(19)			
103	133	3.99	0	0.00	Negative	-229	0	-220.40	A*80:01				A80			
85	141	3.98	0	0.00	Negative	-202	0	-186.18		B*15:02		Bw6		B75(15)		
73	154	3.76	0	0.00	Negative	-184	0	-154.36		B*38:01		Bw4		B38(16)		

Reviewer Comment:

Date: Completed By:

Date: Approved By:

Date: Reviewed By:

Supplementary Figure S1

Before transplantation, 9 kidneys were subjected to Normothermic Machine Perfusion (NMP). NMP is an (experimental) organ-preservation technique in which explanted kidneys are connected to a machine which pumps a 37 ^{0}C preservation fluid through the organ. Because of this, the (cells of) the kidney become metabolically active, and excrete/release various components into the perfusion fluids, including EVs [1]. This allows the analysis of kidney-derived EVs without interference of EVs from other sources. Perfusion fluids were labeled with 200 ng/mL anti-HLA-A3-BV421 (clone GAP-A3, 200 µg /mL, BD Biosciences, New York, USA) and either 200 ng/mL anti-CD9-APC (clone HI9a, 6 µg/mL, Biolegend, San Diego, USA) or 6.6 µg/mL anti-CD63-APC (clone H5C6, 200 µg /mL, Biolegend, San Diego, USA).

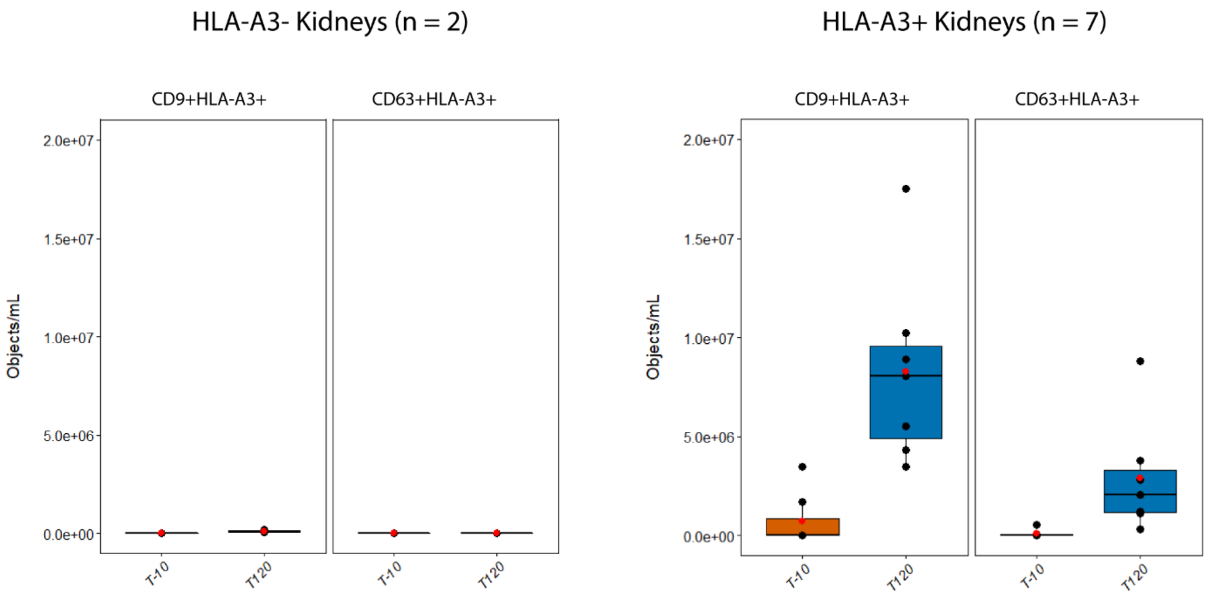

Supplementary Figure S1 – Co-localization of HLA-A3 with CD9 and CD63. Nine kidneys (2x HLA-A3-, 7x HLA-A3+) were subjected to NMP. Perfusate samples taken before (T-10) and 2 hours after (T120) NMP were labelled with anti-CD9 or anti-CD63 in combination with anti-HLA-A3. In perfusion fluids of HLA-A3+ kidneys, an approximate 3-fold higher concentration of CD9+HLA-A3+ EVs was found compared to CD63+HLA-A3+ EVs.

Reference

1. Woud WW, Arykbaeva AS, Alwayn IPJ, Baan CC, Minnee RC, Hoogduijn MJ, et al. Extracellular Vesicles Released During Normothermic Machine Perfusion are Associated with Human Donor Kidney Characteristics. Transplantation. 2022.
